# Time Is Bone: Missed Opportunities for Secondary Prevention After a Hip Fracture

**DOI:** 10.3390/jcm14165816

**Published:** 2025-08-17

**Authors:** Ioannis I. Daskalakis, Johannes D. Bastian, Theodoros H. Tosounidis

**Affiliations:** 1 Department of Orthopaedic Surgery, Medical School, University Hospital of Heraklion, University of Crete, 71110 Heraklion, Greece; g_dask@hotmail.gr; 2 Department of Orthopaedic Surgery and Traumatology, Inselspital, Bern University Hospital, University of Bern, 3010 Bern, Switzerland

**Keywords:** hip fracture, osteoporosis, surgical procedures, secondary prevention, systematic review, bone density

## Abstract

**Background/Objectives:** Early evaluation and treatment of osteoporosis following a hip fracture is of paramount importance for secondary fracture prevention. Nevertheless, the extent to which osteoporosis management is documented in studies reporting on operatively treated hip fractures in elderly patients is unclear. This study is the first systematic review aiming to investigate and summarize the reporting of osteoporosis management in studies with operatively treated hip fractures in elderly patients. **Methods:** This systematic review was conducted in compliance with PRISMA guidelines. A comprehensive search within the last decade of PubMed, Embase, Cochrane Library, Web of Science, and Ovid was performed. Studies reporting on operatively treated hip fractures in patients older than 65 years of age were included. Two reviewers independently screened the studies and performed data extraction. A subsequent descriptive synthesis was performed. **Results:** Eighty-six (86) articles were included in this study. Osteoporosis management was reported in only twelve (12) studies. Only six (6) of them were conducted in institutions with established orthogeriatric care. **Conclusions:** Osteoporosis management is underreported in studies involving operatively treated hip fracture patients. This reflects a significant gap in the overall reporting of secondary fracture prevention actions. Consequently, we advocate for both (a) clinical vigilance for adherence to best practice related to osteoporosis management after the first hip fracture and reporting of the results and (b) the research focusing on the outcomes of secondary fracture prevention efforts.

## 1. Introduction

Hip fractures constitute a major global healthcare problem, particularly for elderly adults, due to their high rates of morbidity, mortality, and healthcare costs [1], ranking among the 10 most common causes of global disability [2]. Patients with a fragility fracture deprived of appropriate subsequent osteoporosis management have more than an 85% increased risk of suffering a secondary fracture [3], with secondary fractures occurring in approximately one-third and three-quarters of patients within one and five years [4]. Multiple clinical guidelines strongly recommend the early assessment and initiation of anti-osteoporotic therapy following a hip fracture [5]. Nevertheless, osteoporosis treatment rates remain low, and less than a third of hip fracture patients receive appropriate anti-osteoporotic medication after a hip fracture [6].

It was our impression that the documentation of the overall handling of osteoporosis in studies reporting on the surgical management of hip fractures in elderly patients was very poor in the contemporary literature. This lack of such reporting significantly impedes our understanding of real-world practices and limits efforts to improve secondary prevention strategies. The primary aim of this systematic review is to assess the extent to which osteoporosis management is reported in studies involving operatively treated older hip fracture patients. We sought to document the following three parameters of osteoporosis evaluation and management: (a) laboratory work-up of osteoporosis, (b) initiation of therapy, and (c) referral for follow-up care.

## 2. Material and Methods

### 2.1. Search Strategy

**Figure 1:**
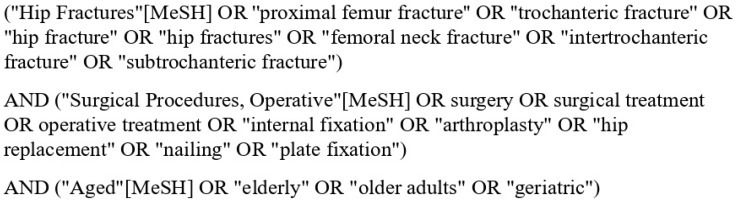
Search strategy adopted throughout the databases; PubMed is shown in this instance.

This systematic review was conducted in accordance with the Preferred Reporting Items for Systematic Reviews and Meta-Analyses (PRISMA) guidelines [7]. A comprehensive literature search was conducted across PubMed, Embase, Cochrane Library, Web of Science, and Ovid, from March 2015 to March 2025. Search terms included (“Hip Fractures” or ‘’proximal femur fracture’’ or ‘’trochanteric fracture’’ or “femoral neck fracture” or “intertrochanteric fracture” or “subtrochanteric fracture”) and (“Surgical Procedures, Operative” or surgery or surgical treatment or operative treatment or “internal fixation” “arthroplasty” or “hip replacement” OR “nailing” or “plate fixation”) and “elderly” or “older adults” or “geriatric”. The search strategy is shown in Figure 1, using PubMed as an example.

### 2.2. Inclusion and Exclusion Criteria

Inclusion criteria: studies involving patients aged ≥65 years who received surgical treatment for hip fractures. Randomized controlled studies and prospective and retrospective cohort studies were included.

Exclusion criteria: (1) studies that did not directly assess outcomes following the operative management of hip fracture, (2) studies that included patients younger than 65 years, (3) studies with unextractable data, (4) studies specifically investigating the effectiveness of osteoporosis management mechanisms, such as the fracture liaison service (FLS) or other specific osteoporosis management systems, (5) and non-English-language publications.

### 2.3. Study Selection

Two independent researchers (ID and JD) screened for potentially relevant studies by reviewing the titles and abstracts. The full texts were then screened further according to the inclusion and exclusion criteria. Any uncertainty was solved by a third researcher (TT).

### 2.4. Data Extraction

Two researchers (ID and JD) independently extracted and recorded the following information from the enrolled studies: author, year, country, sample size, sex, mean age, type of study, outcomes of interest, level of evidence, and report of osteoporosis management. Two investigators independently collected the data, and any discrepancies were resolved by a joint discussion or by a third investigator’s advice (TT).

### 2.5. Data Synthesis and Analysis

A narrative synthesis of the findings was conducted. Due to heterogeneity in study designs, reporting methods, and time frames, a meta-analysis could not be performed. Descriptive statistics were used to summarize the rates of osteoporosis management reporting across studies.

## 3. Results

### 3.1. Flow and Characteristics of Included Studies

**Figure 2:**
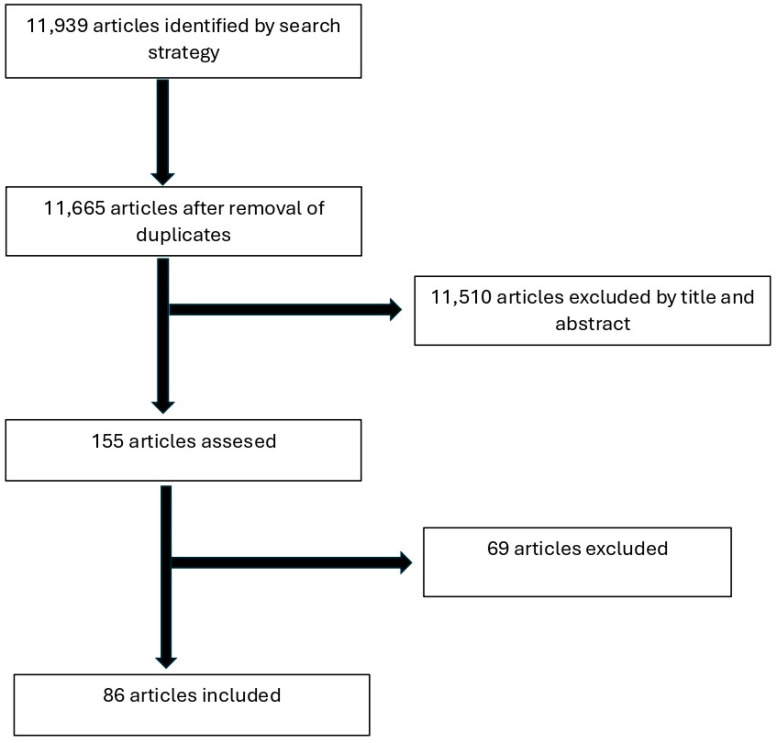
Flow diagram of included studies.

Eighty-six (86) articles [8,9,10,11,12,13,14,15,16,17,18,19,20,21,22,23,24,25,26,27,28,29,30,31,32,33,34,35,36,37,38,39,40,41,42,43,44,45,46,47,48,49,50,51,52,53,54,55,56,57,58,59,60,61,62,63,64,65,66,67,68,69,70,71,72,73,74,75,76,77,78,79,80,81,82,83,84,85,86,87,88,89,90,91,92,93] were included in this study. A total of 22 articles (25%) were published in orthopedic surgery, 4 (5%) in general surgery, and 12 (14%) in trauma journals. Forty-five (52%) were published in journals with other primary areas of focus. Seventy-two articles (84%) listed an orthopedic surgeon as either the first or last author. Seventy (70) studies (82%) were conducted in orthopedic surgery departments. The flow diagram of the included studies is shown in Figure 2. The characteristics of the studies are summarized in Table 1.

### 3.2. Reporting of Osteoporosis Management

**Figure 3:**
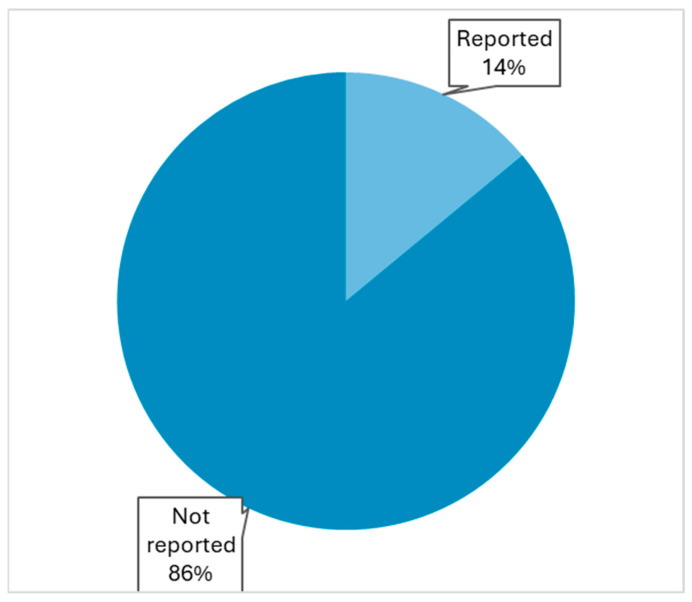
Reporting rates of osteoporosis management.

Only 12 (14%) of the total 86 studies reported some facet of osteoporosis management following hip fracture surgery (Figure 3, Table S1). More specifically, the following parameters were reported: Screening for osteoporosis risk factors in 2 studies [36,48], blood test assessment in 3 studies [20,48,53], bone mineral density (BMD) testing in 5 studies [20,53,71,72,92], supplementation with calcium and/or vitamin D in 6 studies [20,53,70,72,92], initiation of anti-osteoporotic medication (e.g., bisphosphonates) in 7 studies [20,53,59,61,64,72,92], referral to fracture liaison services in 1 study [20], and referral to general practitioner reported in 1 study [36].

### 3.3. Trends in Reporting

Half (6) of the 12 studies that reported on osteoporosis management originated from institutions with established orthogeriatric programs [20,36,48,59,64,72]. Despite this observation, no clear geographic trend was identified, due to the limited number of reporting studies, combined with variability in study design, population characteristics, and healthcare system structures. Notably, the studies by Civinini et al. [20] and Prieto-Alhambra et al. [53] provided the most comprehensive and detailed reports of osteoporosis management, as specific diagnostic pathways were described, along with their results and final treatment rates.

### 3.4. Non-Reporting Studies

In the remaining 74 studies, representing 86.1% of the total included studies, there was an absence of reporting related to osteoporosis diagnosis, management, or follow-up care. These studies primarily concentrated on evaluating clinical outcomes following surgical intervention. Key outcome measures consistently reported included surgical success rates, patient mortality, functional recovery parameters, such as mobility scores or activities of daily living, and the incidence of postoperative complications, including infections and implant failures.

Secondary fracture prevention strategies were not included in the reported protocols or outcomes of the studies. There was no documented assessment of bone mineral density testing, osteoporosis screening, pharmacologic treatment initiation, or referral to osteoporosis management services following discharge. Furthermore, follow-up care was predominantly described in terms of rehabilitation progress and complication management, without a description of secondary fracture prevention measures.

## 4. Discussion

Hip fractures in older adults represent a major cause of morbidity and mortality and are often the result of underlying osteoporosis. Even though the guidelines recommend systematic evaluation and treatment of osteoporosis after a fragility fracture [5,94], this is not always the case and consequently, a major gap in the overall management of these devastating injuries is observed. The goal of this systematic review was to assess the frequency of any form of osteoporosis management report in studies involving patients undergoing surgical treatment for hip fractures, to offer better comprehension of the current status of secondary fracture prevention efforts reporting rates in this high-risk population.

According to our study results, only 12 of the total 86 studies included in this review (14%) reported some aspect of osteoporosis management. Reports included different aspects of osteoporosis management, such as assessment of risk factors, bone mineral density testing, supplementation with calcium and vitamin D, initiation of anti-osteoporotic treatment, or a combination of these. A detailed description of the osteoporosis pathway was provided in three studies. These findings indicate that when osteoporosis management is not involved in the research question, it is typically not reported, even in studies involving hip fractures, which are the most significant osteoporotic fractures. The above findings demonstrate that the majority of the contemporary clinical research on surgical hip management documents insufficiently every facet of secondary fracture prevention. Six of twelve studies that reported osteoporosis management originated from institutions with orthogeriatric care programs, demonstrating that integrated care programs can significantly improve awareness of secondary fracture prevention not only through clinical interventions but also by accurate reporting of osteoporosis management, and therefore directly contribute to focused efforts and strategic initiatives.

Our findings are consistent with the previous research that shows persistent undertreatment of osteoporosis in fracture care. Low rates of osteoporosis treatment initiation after hip fractures have been reported in a substantial number of studies [95]. Kim et al. reported that less than a third of hip fracture patients receive appropriate anti-osteoporotic medication [6], a finding that has been confirmed in multiple studies [96,97]. Underreporting of osteoporosis management is not only a clinical issue but also a significant research gap, particularly in the orthopedic trauma literature, where secondary fracture prevention is often overlooked unless it constitutes the primary focus of the study. Insufficient secondary fracture prevention is most often the result of fragmented care and insufficient interdisciplinary collaboration [3,98]. Integrated care programs, such as orthogeriatric care, have been associated with higher rates of diagnosing osteoporosis, initiation of calcium and vitamin D supplements, and anti-osteoporosis medication [99]. The results of our review are in line with this finding, as half of the studies (6 of 12) that reported osteoporosis management originated from institutions with orthogeriatric care, demonstrating that integrated care models can not only enhance the clinical management of osteoporosis but also improve reporting rates and advance research efforts

The absence of osteoporosis reporting in surgically treated hip fracture cohorts possibly reflects missed opportunities to reinforce secondary prevention as a standard of care. Our study results identify clinical, but mostly research-related gaps and opportunities. From a clinical perspective, the need for integrated care models, such as the FLS and orthogeriatric care, which have demonstrated success in improving treatment rates and reducing re-fracture risk [100], is highlighted by the fact that osteoporosis management is more accurately reported in studies originating from orthogeriatric care units. From a research perspective, greater accountability regarding secondary fracture prevention is required: cohort studies involving fragility fractures should consistently report on osteoporosis assessment and management, even when it does not constitute the primary endpoint. Adopting this strategy would facilitate the full integration of secondary fracture prevention into orthopedic practice and establish it as a key quality indicator.

This review has some limitations. Analysis was limited to descriptive statistics without formal meta-analysis due to the heterogeneity of the included studies. Furthermore, it is possible that osteoporosis management has been performed but not explicitly reported in some studies, leading to potential underestimation. Finally, only English-language articles were included, possibly excluding relevant work from other regions. Nevertheless, to the best of our knowledge, this is the first and only effort aiming to investigate the reporting rates of osteoporosis management in studies involving patients with hip fractures.

## 5. Conclusions

This study is the first systematic review in the literature to provide evidence that osteoporosis management is not adequately reported in the research involving surgically treated hip fractures, when osteoporosis interventions are not included in the study objectives. This finding reveals a significant research gap in documenting secondary prevention in this high-risk population. Based on the aforementioned evidence, we advocate that ongoing and future research related to hip fractures should invariably integrate osteoporosis and secondary fracture management reporting and documentation, with specification of certain parameters and outcomes, regardless of the study’s focus. Additionally, continuous effort is necessary to promote integrated care models, such as the FLS and orthogeriatric care, and report their outcomes to improve secondary fracture prevention.

## Figures and Tables

**Table 1:** Characteristics of included studies (BMD: bone mineral density, DXA: dual-energy X-ray absorptiometry, and NR: not reported).

Author	Year	Country	Sample Size	Sex (Male:Female)	Mean Age	Type of Study	Outcomes of Interest	Level of Evidence	Osteoporosis Management
Abdalattif et al. [8]	2023	UK	148	60:88	78.5	Retrospective	Non-union and AVN incidence	III	NR
Adler et al. [9]	2023	USA	33,142	9180:23,962	86.1	Retrospective	Mortality, delirium, hospice services, SNF admission, nursing home admissions	III	NR
Adulkasem et al. [10]	2021	Thailand	221	36:185	84	Retrospective	Pre-injury New Mobility Score, score at the time of discharge, one year postoperative score	III	NR
Andriollo et al. [11]	2023	Italy	86	20:86	87.4	Retrospective	Charlson Comorbidity Index, Barthel index, Koval Grade, Mental Score, subsequent hospitalizations for surgical operations relating to the operated hip	III	NR
Arraut et al. [12]	2022	USA	110	23:87	Younger cohort: 67.69 Older cohort: 85.12	Retrospective matched cohort	Discharge disposition, 90-days postoperative outcomes	III	NR
Barışhan et al. [13]	2018	Turkey	38	13:25	THA: 73.6 HA: 76.9	Retrospective	Clinical outcomes and mortality	III	NR
Bayon-Calatayud et al. [14]	2018	Spain	50	11:39	84.1	Prospective cohort	Barthel score	II	NR
Bi et al. [15]	2023	China	72	28:44	68.59	Retrospective	Perioperative indicators, functional outcome (Harris hip score), complications one year postoperatively	III	NR
Bigoni et al. [16]	2020	Italy	244	79:65	80	Retrospective	Mortality rate, complications, reoperation rate	IV	NR
Blauth et al. [17]	2021	USA	281	74:207	Geriatric center: 81.9 Usual care: 83.9	Prospective multicenter	Major adverse effects, mortality	II	NR
Bűcs et al. [18]	2021	Hungary	94	35:59	79	Prospective cohort	Perioperative indicators, mobilization, length of stay, functional outcome (Harris hip score)	II	NR
Chatterji et al. [19]	2022	India	40	16:24	THA: 70.28 HA: 68.5	Prospective cohort	Functional outcome (Harris hip score)	II	NR
Chen et al. [73]	2020	China	130	49:81	HA: 78.3 IF: 75.1	Retrospective cohort	Incidence of surgical complications and reoperation, mortality, hip joint function at the last follow-up, perioperative parameters	III	NR
Chen et al. [70]	2019	China	313	92:221	77.6	Retrospective cohort	1-year mortality	III	NR
Chen et al [69]	2024	China	458	121:276	No fracture: 71 Contralateral hip fracture: 84	Retrospective	Analysis of contralateral hip fracture risk factors	III	Administration of calcitriol and calcium
Cho et al. [71]	2016	China	194	69:125	DHS: 84.2 PFNA: 81	Retrospective	Operative time, blood loss, walking ability, Barthel index, fracture union, proximal femur shortening, complications	III	BMD testing (DXA)
Civinini et al. [21]	2019	Italy	677	210:467	84.5	Prospective cohort	Mortality, return to daily activities, quality of life, adherence to re-fracture prevention programs	II	FLS, BMD testing and evaluation of fall and fracture within 3 months for 434 (66%) eligible patients, prescription of specific drugs and calcium ± vitamin D supplementation in 342 (78.8%) patients
Dayama et al. [21]	2016	USA	3121	938:2183	77.34	Retrospective registry study	30-day morbidity and mortality	III	NR
Ekinci et al. [22]	2020	Turkey	308	81:227	BHA: 78.4 PFN: 77.7	Prospective cohort	Singh Index, functional outcome (Harris hip score)	II	NR
Fernandez et al. [23]	2023	France	181	40:41	82.5	Retrospective cohort	Fixation failure rate at 3 and 6 months, quality of life, Parker mobility score, Harris hip score	III	NR
Guo et al. [24]	2019	China	17	4:10	67.6	Retrospective cohort	Fracture union, complications, functional outcome (Harris hip score)	III	NR
Huang et al. [25]	2020	China	202	53:149	86	Retrospective	Operative duration, blood loss, time of weight-bearing after operation, complications, functional outcome (Harris hip score)	III	NR
Garcia-Barreiro et al. [26]	2023	Argentina	375	58:317	86.1	Retrospective	Functional outcome (Parker mobility score)	III	NR
Gilmore et al. [89]	2024	UK	502	137:335	80.1	Retrospective	30-day and 1-year mortality	III	NR
Gölge et al. [27]	2016	Turkey	202	90:112	HA: 78.6 PFN: 75.7	Retrospective	Mortality	III	NR
Iorio et al. [28]	2019	Italy	60	25:35	HA: 83 THA: 82	Prospective randomized	Dislocation rate at a minimum follow-up of 1 year, reoperation rate, time to surgery, surgical time, length of hospital stay, 30-day and 1-year mortality	I	NR
Jonas et al. [29]	2015	UK	132	22:88	HA: 79 THA: 78	Retrospective	Functional outcome (Oxford Hip Score), quality of life (SF-36), complications	III	NR
Ju et al. [30]	2020	China	73	16:57	68.22	Retrospective	Necrosis of femoral head, functional outcome (Harris Hip score)	III	NR
Karaali et al. [31]	2021	Turkey	129	44:85	Metaphyseal fixed HA: 78 Diaphyseal fixed HA: 79	Retrospective	2-year functional outcome (Harris hip score, Parker Mobility Score), mortality	III	NR
Kawaji et al. [32]	2015	Japan	42	9:33	Asian IMHS: 78.4 Conv. IMHS:82.3	Retrospective	Walking ability, complications	III	NR
Khan et al. [33]	2021	India	88	43:45	Unipolar group: 67.2 Bipolar group: 66.1	Prospective randomized	Radiological outcome, functional outcome (Harris hip score)	I	NR
Knauf et al. [34]	2019	Germany	395	109:286	81	Prospective	Mortality	II	NR
Koyuncu et al. [35]	2015	Turkey	152	67:85	76	Prospective	Clinical and radiological outcomes, complications	II	NR
Kusen et al. [36]	2019	Switzerland	322	87:225	2013: 86 2016: 85	Retrospective and prospective cohort	Peri-operative data, postoperative outcomes and complications	III	Osteoporosis screening, referral to general practitioner
Kusen et al. [37]	2021	Switzerland	752	203:549	86	Prospective cohort	Mortality, complications, time to surgical intervention, hospital length of stay	II	BMD testing (DEXA scan)
Laubach et al. [38]	2021	Germany	1727	584:1143	Internal fixation: 81 Arthroplasty: 83	Retrospective	Μobility, residential status, reoperation rate, HRQoL, mortality	III	NR
Lee et al. [72]	2021	Canada	212	49:163	Preintervention group: 84 Postintervention group: 85	Retrospective	Length of stay, incidence of delirium	III	Osteoporosis assessment, prescription of calcium and vitamin D
Lee et al. [88]	2019	South Korea	234	49:185	80.6	Prospective cohort	Clinical outcomes (Koval’s categories for walking ability), radiographic outcome	II	NR
Leonardsson et al. [39]	2016	Sweden	2128	559:1569	85	Retrospective	Quality of life, pain	III	NR
Li et al. [40]	2018	China	43	17:26	76.5	Retrospective	Functional outcome (Harris hip score), mobility (TUG test, Parker score	III	NR
Liang et al. [41]	2022	China	87	32:55	PFNA: 86.1 External fixation: 85.6	Prospective randomized	Functional and radiographic outcomes, complications	I	NR
Liu et al. [42]	2020	China	156	75:81	HA: 78.1 Fixation: 76.2	Prospective cohort	Quality of life, cost-effectiveness	II	NR
Lu et al. [43]	2016	China	78	20:68	Fixation: 85.85 HA: 86.24	Prospective randomized	Operative outcome, hip functions	I	NR
MacLellan et al. [44]	2024	Canada	659	193:489	82.8	Retrospective	Length of stay, discharge location, mortality	III	NR
Mallick et al. [45]	2019	UK	168	45:122	82	Retrospective	Complications, mortality	III	NR
Moaz et al. [46]	2024	Pakistan	102	56:46	65.4	Prospective cohort	Functional outcome (Harris hip score)	II	NR
Moore et al. [47]	2023	Ireland	7.109	2.203/4.906	81	Retrospective registry study	Post-op mobility, 7-day and 14-day inpatient mortality, discharge destination	III	NR
Morris et al. [48]	2023	New Zealand	181	46:135	2011 cohort: 84.2 2017 cohort: 82.6	Retrospective	Length of stay, postoperative complications, 30-day and 1-year mortality	III	Assessment of risk factors, metabolic blood tests screening
Mukka et al. [49]	2020	Sweden	235	75:160	83	Prospective pilot study	Functional outcome (Harris hip score, WOMAC), pain (PNRS)	II	NR
Okano et al. [50]	2017	Japan	16	2:14	86.9	Retrospective	Implant-related complications	III	NR
Park et al. [93]	2022	Korea	34	9:25	82.68	Prospective cohort	Operation time, bleeding, time to walk, complications	II	NR
Peddamadyam et al. [51]	2024	India	20	8:12	71.65	Prospective cohort	Clinical outcome, complications	II	NR
Prestmo et al. [52]	2015	Norway	397	104:293	83	Prospective randomized study	Mobility at 4 months (SPPB)	I	NR
Prieto-Alhambra et al. [53]	2018	Spain	997	232:765	83.6	Prospective cohort	In-patient care, complications, and 4-month mortality	II	Osteoporosis not assessed for 23.6%, assessed but treatment unnecessary for 20.5%, 14.9% awaiting an osteoporosis clinical assessment, 3.2% discharged pending a DXA scan, prescription of anti-osteoporotic treatment at 21.4%
Ratanpai et al. [54]	2025	India	110	37:73	71.3	Prospective cohort	Functional outcome (modified Harris hip score and Oxford Hip Score), 1-year mortality	II	NR
Regis et al. [55]	2023	Italy	139	41:98	Uncemented HA: 80.1 Cemented HA: 84.3	Retrospective cohort	Surgical time, overall perioperative complication rate	II	NR
Roll et al. [90]	2018	Germany	1015	275:740	83.2	Prospective cohort	Procedural and patient outcome parameters	II	NR
Sadeghi et al. [56]	2020	USA	5526	1658:3868	Long nail: 80.6 Short nail: 81.2	Retrospective cohort	Risks of all-cause revision and revision for periprosthetic fracture	III	NR
Salvesen et al. [57]	2025	Norway	398	116:282	83	Prospective cohort	SPPB, EuroQol-5-dimension and5-level, Barthel index, Lawton and Brody Instrumental Activities of Daily Living, Lawton and Brody Self-Maintenance Scale, readmission and mortality	II	NR
Sanderson-Jerome [58]	2024	TTO	30	13:17	82.0	Prospective cohort	Time to surgery (lead time), complications by Clavien–Dindo score, hospital length of stay and mortality, and costs of hospitalization	II	NR
Schoenberg et al. [59]	2021	Germany	9780		84.4	Retrospective registry	Rate of readmission, rate of re-surgery, anti-osteoporotic therapy, housing, mortality, walking ability, and quality of life 120 days post-surgery	III	Initiation of anti-osteoporotic medication
Schuijt et al. [60]	2020	Netherlands	806	579	85	Retrospective cohort	Postoperative complications, patient mortality, time spent at the emergency department, time to surgery, hospital length of stay	III	NR
Sun et al. [62]	2023	China	701	201:500	Early surgery: 80.1 Late surgery: 79.56	Retrospective	Quality of life, complications	III	NR
Sun et al. [61]	2024	China	669	179:490	72.49	Multicenter cross-sectional study	Current status of postoperative care for elderly osteoporotic fracture patient	II	Osteoporosis treatment for 303 (45.3%) patients after surgery
Sun et al. [63]	2024	China	40	10:30	ITF: 72.8 PFNA: 73.7	Retrospective cohort	Surgical and rehabilitation data, functional outcome (Harris hip score), quality of life (SF-36), complications	III	NR
Solberg et al. [64]	2023	Norway	516	146:370	84	Prospective cohort	Comparison of two orthogeriatric care models	II	Treatment with anti-osteoporosis drugs in hospital: 70%
Sniderman et al. [65]	2023	Canada	150	34:116	HA: 83.2 ORIF: 84	Retrospective cohort	Blood loss, complications	III	NR
Song et al. [66]	2020	China	94	62:32	Control group: 70.6 Delayed group: 73	Prospective cohort	Functional outcome (Harris hip score), quality of life	II	NR
Sundkvist et al. [67]	2024	Sweden	291	139:152	82	Multicenter prospective cohort	Treatment failure, reoperations, mortality rate at 30 days, 90 days, and 1 year	II	NR
Tan et al. [68]	2017	Singapore	2029	518:1511	78.8	Retrospective	Complications, ambulatory status at discharge, length of hospital stay	III	NR
Takahashi et al. [74]	2020	Japan	228	56:172	85	Retrospective cohort	Patients’ ambulation ability before injury, at discharge, and 6 months after injury (Functional Ambulation Category), presurgical duration, length of hospital stay, time until beginning to walk using parallel bars, complications affecting treatment, mortality rate	III	NR
Tian et al. [75]	2020	China	90	48:42	70.5	Prospective cohort	Functional outcome (Harris hip score), complications	II	NR
Tilaveridis et al. [76]	2023	Greece	118		82.7	Prospective cohort	Radiographic outcome, complications	II	NR
Tol et al. [77]	2017	Netherlands	252	47:205	81.1	Prospective randomized	Functional outcome, (modified Harris hip score), postoperative complications, intra-operative data	I	NR
Uzel et al. [78]	2024	Turkey	53,495	35.841:17.654	86	Retrospective registry	Complication, mortality	III	NR
Vasu et al. [79]	2018	India	60	34:26		Prospective cohort	Association of modified frailty index (MFI) with 90-day mortality	II	NR
Vigano et al. [82]	2023	Italy	79,120	17,040:54,877	84	Retrospective registry	Fracture incidence, epidemiology, mortality, cost	III	NR
Wang et al. [83]	2023	China	644	174:470	81.66	Retrospective	1-year mortality, major complications, and AKI by BMI category	III	NR
Wang et al. [82]	2022	China	761	247:514	81.23	Retrospective	Length of hospital stay, postoperative complications, 30-day and one-year mortalities, one-year functional status	III	NR
Wignadasan et al. [84]	2024	UK	335	105:224	Cemented: 84.7 Uncemented: 85.9	Retrospective	Length of hospital stay, discharge destination, morbidity, mortality, theater time, cost	III	NR
Xu et al. [85]	2025	China	300	161:139	85.88	Prospective cohort	Fracture reduction, Harris hip score at 1 and 6 months after surgery, failure of internal fixation	II	NR
Yan et al. [86]	2024	China	80	23:57	80.5	Retrospective	Torn conjoined tendons, dislocation and complication rate	III	NR
Yin et al. [87]	2024	China	141	50:91	DAA: 73.98 PLA: 76.72	Retrospective	Hospitalization days, VAS score, Harris hip score at one month and six months, incidence of complications, revision rate, one-year survival rate, patient satisfaction	III	NR
Yu et al. [80]	2016	China	117	67:80	PFNA-II: 74.2 IT group: 75.2	Prospective	Intraoperative variables, postoperative complications	II	NR
Zhou et al. [91]	2020	China	224	114:110	68.52	Prospective cohort	Functional outcome (Harris hip score), rates of revision, loosening, periprosthetic fracture, dislocation	II	NR
Zhou et al. [92]	2019	China	108	63:45	75.3	Prospective cohort	Operation time, intraoperative bleeding, immobilization duration, hospitalization time, Harris hip score, postoperative complications	II	BMD testing (DXA), initiation of alendronate and vitamin D3

## Data Availability

The raw data supporting the conclusions of this article will be made available by the authors on request.

## Supplementary Materials

The following supporting information can be downloaded at: https://www.mdpi.com/article/10.3390/jcm14165816/s1, Table S1: summarizes the studies that reported osteoporosis management.
