# Supplementary material for: Time Is Bone: Missed Opportunities for Secondary Prevention After a Hip Fracture
Source: J Clin Med. 2025 Aug 17;14(16):5816. doi: 10.3390/jcm14165816 (PMC12386996; doi:10.3390/jcm14165816)
Supplement: Supplementary file 1 [file jcm-14-05816-s001.zip › jcm-3803009-supplementary.pdf]

Author	Year	Country	Sample size	Sex (male: female)	Mean age	Type of study	Outcomes of interest	Level of evidence	Osteoporosis management
Chen et al	2024	China	458	121:276	No fracture: 71 Contralateral hip fracture: 84	Retrospective	Analysis of contralateral hip fracture risk factors	III	Administration of calcitriol and calcium
Cho et al	2016	China	194	69:125	DHS:84.2<br>PFNA: 81	Retrospective	Operative time, blood loss, walking ability, Barthel index, fracture union, proximal femur shortening, complications	III	BMD testing (DXA)
Civinini et al	2019	Italy	677	210:467	84.5	Prospective cohort	Mortality, return to daily activities, quality of life, adherence to re-fracture prevention programs	II	FLS, BMD testing and evaluation of fall and fracture within 3 months for 434 (66%) eligible patients, prescription of specific drugs and calcium ± vitamin D supplementation<br>in 342 (78.8%) patients
Kusen et al	2019	Switzerland	322	87:225	2013:86<br>2016:85	Retrospective and prospective cohort	Peri-operative data, post-operative outcomes and complications	III	Osteoporosis screening, referral to general practitioner
Kusen et al	2021	Switzerland	752	203:549	86	Prospective cohort	Mortality, complications, time to surgical intervention, hospital length of stay	II	BMD testing (DEXA scan)
Lee et al	2021	Canada	212	49: 163	Preintervention group: 84<br>Postintervention group:85	Retrospective	Length of stay, incidence of delirium	III	Bone health assessment, prescription of calcium and Vitamin D

Morris et al	2023	New Zealand	181	46:135	2011 cohort: 84.2<br>2017 cohort: 82.6	Retrospective	Length of stay, postoperative complications, 30-day and 1-year mortality	III	Assessment of risk factors, metabolic blood tests screening
Prieto-Alhambra et al	2018	Spain	997	232:765	83.6	Prospective cohort	In-patient care, complications, and 4-month mortality	II	Bone health not assessed for 23.6%, assessed but treatment unnecessary for 20.5%, 14.9% awaiting a bone health clinical assessment, 3.2% discharged pending a DXA scan<br>Prescription of antiosteoporotic treatment at 21.4%
Schoenberg et al	2021	Germany	9780		84.4	Retrospective registry	Rate of readmission, rate of re-surgery, anti-osteoporotic therapy, housing, mortality, walking ability, and quality of life 120 days post-surgery	III	Initiation of antiosteoporotic medication
Sun et al	2024	China	669	179:490	72.49	Multicenter cross-sectional study	Current status of postoperative care for elderly osteoporotic fracture patient	II	Osteoporosis treatment for 303 (45.3%) patients<br>after surgery
Solberg et al	2023	Norway	516	146:370	84	Prospective cohort	Comparison of two orthogeriatric care models	II	Treatment with antiosteoporosis drugs in hospital: 70%
Zhou et al	2019	China	108	63:45	75.3	Prospective cohort	Operation time, intraoperative bleeding, immobilization duration, hospitalization time, Harris Hip score,	II	BMD testing (DXA), initiation of alendronate and Vitamin D3

		postoperative complications	

Table S1: Summary of studies reporting osteoporosis management
